# Right atrial pressure alterations during echocardiography-guided-catheterization predict tricuspid valvular impairment: a novel method for the creation of a rabbit model of *Staphylococcus aureus* endocarditis

**DOI:** 10.1186/1476-7120-12-21

**Published:** 2014-06-20

**Authors:** Mei-lian Wang, Ying Zhang, Miao Fan, Ya-jun Guo, Wei-dong Ren, En-jie Luo

**Affiliations:** 1 Department of Microbiology and Parasitology, College of Basic Medical Sciences, China Medical University, No. 92 Beier Road, Shenyang 110001, Heping District, China; 2 Department of Sonography, Shengjing Hospital of China Medical University, No. 36 Sanhao Street, Shenyang 110004, Heping District, China; 3 Department of Radiology, The First Affiliated Hospital of Sun Yat-sen University, Guangzhou 510080, China

**Keywords:** Infective endocarditis, Echocardiography, Catheterization, Right atrial pressure, Animal model

## Abstract

**Background:**

We previously reported the use of a catheter system to damage the tricuspid valve and create infectious endocarditis (IE) in an animal model. The current study aims to create a faint IE model suitable for antibiotic prophylaxis using a low bacterial inoculum. We also aim to explore a way to quantitatively assess valvular impairment and to predict the success of the IE models during catheterization.

**Methods:**

Ninety rabbits were assigned to two groups according to the density of bacteria inoculated (1 × 10^5^ CFU for Group A and 1 × 10^4^ CFU for Group B). A catheter system consisting of a polyethylene catheter and a guide wire were used to damage the valve. The catheter system was passed through the rabbits’ tricuspid valves under echocardiographic guidance. A pressure transducer was used to assess right atrial pressure (P_RA_) before and just after valvular damage to calculate the pressure alterations (ΔP_RA_). The animals in group A and B were divided into 3 subgroups according to the ΔP_RA_ (0–5 mmHg for Groups A_1_ and B_1_; 5–10 mmHg for Groups A_2_ and B_2_; 10–15 mmHg for Groups A_3_ and B_3_). *Staphylococcus aureus* (ATCC 29213) inoculation was performed 24 hr after cardiac catheterization.

**Results:**

Faint IE was confirmed in 20%, 93.3%, 26.7%, 6.7%, 20%, and 33.3% of the rabbits in Groups A_1_, A_2_, A_3_, B_1_, B_2_, and B_3_, respectively. There was no difference in the LV/RV ratio and V_TR_ of the No-IE, faint-IE, and severe IE animals. Faint IE rabbits had a larger ΔP_RA_ than No-IE rabbits (7.81 ± 1.21 vs. 2.48 ± 1.0, *P* < 0.01, for Group A; 7.60 ± 1.32 vs. 2.98 ± 1.08, *P* < 0.01, for Group B). The ΔP_RA_ of severe IE and faint IE rabbits was significantly different (13.11 ± 1.31 *vs.* 7.81 ± 1.21, *P* < 0.01, for Group A; 12.73 ± 1.44 *vs.*7.60 ± 1.32, *P* < 0.01, for Group B).

**Conclusion:**

ΔP_RA_ could be used to assess valvular impairment. Controlling the value of ΔP_RA_ during catheterization and inoculating of an appropriate dose of bacteria was associated with a successful IE model.

## Background

Infective endocarditis (IE) is a life-threatening disease associated with a high mortality rate [1-3]. It continues to be a challenge in clinical practice. Animal models of IE are widely used to provide a better understanding of the pathogenesis, pathophysiology [4-6], and treatment of intracardiac infections [7-9]. Normally, blood flows smoothly through cardiac valves. If these valves are damaged, the risk of bacterial attachment is increased. There are two main procedures used to create an IE model, damaging a valve and inoculating the host with bacteria. A large inoculum of bacteria is used for most IE models in order to guarantee the successful creation of IE [10-13]. Durack *et al.* injected 10^8^ colony-forming units (CFU) of viridans streptococci IV to produced IE in 100% of the experimental animals [14]. In fact, the dose of bacteria in humans may be 10 to 100 times higher than the lowest infecting dose necessary to produce IE in 90% of the animals. IE models created by injecting a large number of bacteria may be unsatisfactory as they may not be relevant to the human situation. For example, the magnitude of bacteremia observed in humans after certain procedures such as tooth extraction is generally 10^1^ to 10^2^ CFU/ml of blood [15]. A small inoculum similar to that found in humans with low-grade bacteremia is appropriate when the aim of the study is to evaluate the efficacy of a given antibiotic regimen given prior to bacterial challenge [16]. Using animal models with a large bacterial inoculum may require a prolonged administration of higher doses of antibiotics to achieve successful prophylaxis.

Small injected doses of bacteria may produce vegetations too small to be confirmed by echocardiography. This may be more relevant to the human situation [17-19]. We speculate that an appropriate aggravation for valvular damage might guarantee the successful creation of this IE model. We previously reported [20] a rabbit model of right-sided IE using a catheter to damage the tricuspid valve. This model is useful in evaluating the therapeutic effect of different medications on IE. We have improved this model to simulate the clinical setting of antibiotic prophylaxis. We reduced the size of the bacterial inoculation and evaluated valve impairment in this model of IE.

## Methods

### Ethics statement

All animal procedures were approved by the Animal Ethics Committee of China Medical University and were conducted in compliance with institutional regulations.

### Experimental design

We speculated that the density of a bacterial inoculation can affect the development of IE. One hundred animals were used in the study. Ninety were randomly assigned to two groups according to the density of *Staphylococcus aureus* inoculated. 1 × 10^5^ CFU (Group A) or 1 × 10^4^ CFU (Group B) of *S. aureus* was inoculated 24 hr after right heart catheterization. Five animals also had right heart catheterization performed without inoculation (Group C) and five animals were only inoculated with bacteria (Group D).

The extent of damage to the tricuspid valve affected the success rate of IE in the animal models. This might be related to the degree of right atrial pressure change related to instrumentation (ΔP_RA_). We quantified valvular impairment by measuring the ΔP_RA._ ΔP_RA_ was controlled to a predicted range of values when damaging the tricuspid valves. For example, the catheter system was manipulated forward and backward across the tricuspid valve ten times to produce severe damage and once to produce mild damage. The number of times the guide wire was passed through the tricuspid valve led to different values of ΔP_RA_. Animals in Group A and B were divided into three sub-groups related to this effect of the wire on the pressure gradient across the tricuspid valve. The pressure gradients were 0–5 mmHg, 5–10 mmHg, and 10–15 mmHg, respectively for Groups A_1_/B_1_, A_2_/B_2_, and A_3_/B_3_. Each subgroup contained 15 animals. The ratio of left ventricle to right ventricle diameter (LV/RV), and peak velocity of tricuspid valve regurgitation (V_TR_) were evaluated during these procedures.

There were three outcomes in this animal model. Animals developed severe IE with vegetations visualized by echocardiography, faint IE with vegetations too small to be detected by echocardiography but confirmed by macroscopic and histologic examination of the cardiac valves, or did not develop IE by echocardiography or by histologic findings. The ΔP_RA_ and echocardiographic findings just after manipulating the tricuspid valves were used to assess model outcomes.

### Experimental animals

One hundred New Zealand white rabbits (50 males and 50 females), weighing 2–2.5 kg, were obtained from Beijing Animal Institute (Beijing, China). They were kept in the animal facility at China Medical University. The rabbits were maintained in individually ventilated cages and supplied heat-sterilized food and distilled water *ad libitum*.

### Right heart catheterization under echocardiographic guidance

**Figure 1:**
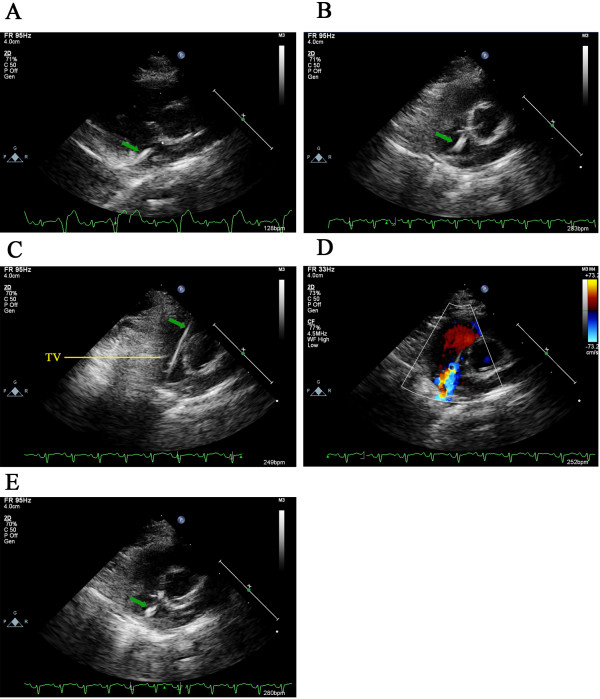
**The process of right-heart catheterization under echocardiographic guidance.** Echocardiography with an aortic short axis view was used to visualize the position of the catheter system. The catheter system was visualized entering the right atrium **(A)**. The guide wire was slowly backed out to connect a pressure transducer to measure pressures in the right atrium. The pressure transducer was removed to allow introduction of the guide wire into the catheter system again. Adjust the direction of the guide wire to advance the catheter system pointing to the tricuspid valve **(B)** and then getting through it **(C)**. The guide wire inside the catheter was then used to damage the tricuspid valve. Color Doppler was in action to visualize tricuspid valve regurgitation **(D)**. The peak velocity was then evaluated by Pulsed Doppler. The catheter system was then repositioned at the entrance of the right atrium **(E)**. The guide wire was removed and a pressure transducer connected to measure the pressure of the right atrium after valvular impairment.

Right heart catheterization was performed under echocardiographic guidance as previously described [20]. Briefly, an incision was made in the left inguinal region after induction of anesthesia with pentobarbital sodium (30 mg/Kg ip). The left femoral vein (LFV) was exposed and dissected to allow introduction of the catheter system. The catheter system consisted of a polyethylene catheter with a steel guide wire. The catheter system was prepared by flushing the external wall and the lumen of the catheter with heparinized sterile saline. The catheter system was introduced into the LFV to the entrance of the right atrium under echocardiographic guidance (Figure 1-A). When the catheter system touched the atrial septum, it was necessary to adjust the direction of the guide wire to advance the system pointing to the tricuspid valve (Figure 1-B) and then across it (Figure 1-C). The guide wire was advanced until about 1 cm of the tip was exposed. The guide wire was moved forward and backward repeatedly over the fragile tricuspid valve to induce damage.

### Evaluation of right atrial pressure (P_RA_)

**Figure 2:**
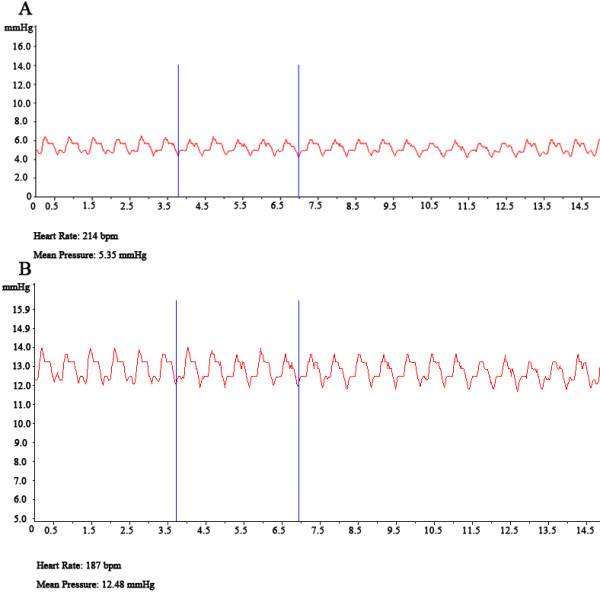
**Right atrial pressure before (A) and just after (B) tricuspid valve impairment.** The mean pressure is displayed at the bottom of the diagram.

The guide wire was slowly backed out after the catheter system was visualized entering the right atrium (Figure 1-A). A 20 ml injection syringe (with 10 ml saline inside) was attached to the end of catheter using a three-way stopcock. Placement was checked using blood aspiration. A pressure transducer (YP-100, Yilian Medicine, Ltd., Shanghai, China) attached to the Biological and Functional Experimental System (BL-420, Taimeng Science technology, Ltd., Chengdu, China) was used to monitor pressure. Data was input into a personal computer using a USB 2.0 data cable. TM_WAVE Bio-signal acquisition and analysis software (version 1.0, Taimeng Science technology, Ltd., Chengdu, China) was used to display the P_RA_ in real-time. Five randomly selected portions of the waveform were used to calculate the mean P_RA_ (Figure 2-A). The pressure transducer was removed to allow introduction of the guide wire into the catheter system and to damage the tricuspid valve, which could be confirmed by the existence of tricuspid regurgitation by Color Doppler (Figure 1-D). The catheter system was then backed out into the right atrium (Figure 1-E). The guide wire was removed and the P_RA_ monitored again (Figure 2-B).

### Echocardiographic measurement

An ultrasound system (Philips iE 33) with a 4–12 MHz transducer was used in this study.

The heart was imaged using a four chamber view immediately after damaging the tricuspid valve. The diameters of the left and right ventricles were measured at the level of atrioventricular valve annulus and at the middle of the two ventricles. The average dimensions of the two ventricles were used to calculate the LV/RV ratio. A four chambers or aortic short axis view was used to confirm the existence of tricuspid valve regurgitation and to acquire the value of V_TR_.

Echocardiography was performed to confirm the existence of cardiac vegetations on aortic short axis and four-chamber views when the rabbits were sacrificed or moribund.

### Production and confirmation of IE

The rabbits were injected with a *S. aureus* (ATCC 29213) suspension (1 × 10^5^ or 1 × 10^4^ CFU) using a marginal ear vein. The body temperature was recorded during the challenge. Blood cultures were performed using intracardiac puncture at the end of the experiment. The presence of IE was confirmed by macroscopic and histologic examination of the cardiac valves. Tricuspid valves were excised and prepared for light microscopy. The specimens were fixed in 10% formalin, embedded in paraffin and cut into 5 μm sections. Sections were stained with hematoxylin & eosin.

### Quantitative microbiologic analysis

Bacterial titers per gram of tissue were determined for cardiac vegetations obtained from rabbits with IE. The tissue fragments were crushed in tryptic soy broth (Sigma, U.S.) and 10-fold serial dilutions were inoculated onto tryptic soy agar plates. Bacterial titers were reported in terms of CFU.

### Statistical analysis

Data was expressed as means ± SD. Differences between multiple means were compared using one-way ANOVA, Tamhane’s T2 test or Bonferroni test when the variance was heterogeneous or homogeneous, respectively. *P* < 0.05 was considered statistically significant. All statistical analyses were performed using commercially available software (SPSS, release 17.0).

## Results

**Figure 3:**
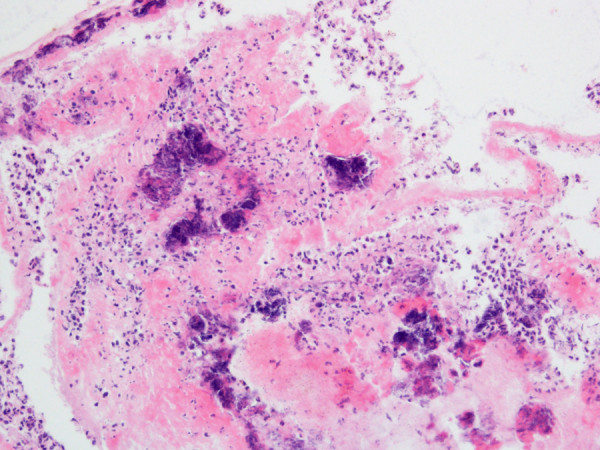
**Histologic examination of the infected tricuspid valve.** Histologic examination showed infectious vegetations with destruction of valvular tissue. Heavy inflammation consisting mostly of neutrophils was seen in the tissue (H&E, 100×).

Only one of the 90 rabbits in experimental groups A and B died prematurely. None of the control animals (Group C and D) died. The body temperature in all inoculated rabbits (Groups A, B, and D) increased to at least 40°C during the 72 hr following inoculation. No discernible difference was observed between rabbits in Group A and B and no febrile response occurred in group C.Macroscopic and histologic examination of the tricuspid valves demonstrated the presence of IE in 42 rabbits, 33 from Group A and 9 from Group B. Vegetations 1–10 mm in size were adherent to the tricuspid valve. The vegetations appeared yellow or gray-white. Histologic examination showed infectious vegetations with destruction of valvular tissue. Heavy inflammation composed mostly of neutrophils was present (Figure 3).

Blood cultures were positive in 29 rabbits from Group A and 3 rabbits from Group B. All except one were sacrificed on day 5. The mean bacterial count of the cardiac vegetations was measured for each rabbit in Group A and B (Table 1).

**Table 1:** Animal death, pathologic findings, blood culture results, and mean bacterial count in cardiac vegetations

**Experimental design**	**Experimental group**		**Control group**	
	**Group A (n = 45)**	**Group B (n = 45)**	**Group C (n = 5)**	**Group D (n = 5)**
Premature death (n)	1	0	0	0
Endocarditis (n)	33	9	0	0
Positive blood culture (n)	29	3	N/A	N/A
MBC in vegetations (×10^4^)	33.24 ± 15.22	9.78 ± 5.66	N/A	N/A

Group A: rabbits underwent both catheterization and inoculation (1 × 10^5^ CFU); Group B: rabbits underwent both catheterization and inoculation (1 × 10^4^ CFU); Group C: rabbits underwent only catheterization; Group D: rabbits only inoculated. *MBC*: Mean bacterial count; *N/A*: Not applicable.

Echocardiographic parameters and ΔP_RA_ were measured after damaging the tricuspid valve. The LV/RV ratio was about 2:1 in all the rabbits in Group A and B. There was no difference in the LV/RV ratio or V_TR_ of the No-IE, Faint-IE, and Severe-IE subgroups. The ΔP_RA_ of the Faint IE rabbits was significantly higher than that of the No-IE rabbits (*P* < 0.01). The ΔP_RA_ of the Severe-IE rabbits was significantly higher than that of the Faint-IE rabbits (*P* < 0.01) (Table 2). The ΔP_RA_ during catheterization was able to predict the success of the IE models.

**Table 2:** Echocardiographic and physiological findings

**Experimental design**	**Group A (1 × 10**^**5**^ **CFU)**			**Group B (1 × 10**^**4**^ **CFU)**		
	**No-IE**	**Faint IE**	**Severe IE**	**No-IE**	**Faint IE**	**Severe IE**
Numbers	12	21	12	36	9	0
LV/RV ratio	2.05 ± 0.23	2.06 ± 0.22	1.94 ± 0.22	2.06 ± 0.35	2.01 ± 0.18	1.96 ± 0.10
V_TR_ (m/s)	0.53 ± 0.26	0.59 ± 0.22	0.66 ± 0.24	0.53 ± 0.25	0.58 ± 0.21	0.62 ± 0.29
ΔP_RA_ (mmHg)	2.48 ± 1.00	7.81 ± 1.21^*^	13.11 ± 1.31^#^	2.98 ± 1.08	7.6 ± 1.32^*^	12.73 ± 1.44^#^

*No-IE*: Did not develop IE by echocardiography or by histologic findings; *Faint IE*: Infectious endocarditis could confirmed by histologic findings but not visualized by echocardiography; *Severe IE*: Infectious endocarditis confirmed by both echocardiography and histologic findings. *LV*: Left ventricle; *RV*: Right ventricle; *V*_*TR*_: Peak velocity of tricuspid valve regurgitation; *ΔP*_*RA*_: Right atrial pressure before and just after valvular impairment was used to calculate alterations of right atrial pressure, indicated as ΔP_RA_.

^*^P < 0.01, Faint IE *vs.* None-IE; ^#^*P* < 0.01, Severe IE *vs.* Faint IE.

Animals in Groups A and B were divided into groups according to ΔP_RA_. Faint IE was confirmed in 20%, 93.3%, 26.7%, 6.7%, 20%, and 33.3% of the rabbits in Group A_1_, A_2_, A_3_, B_1_, B_2_, and B_3_, respectively (Table 3). The Faint-IE model best correlated with a ΔP_RA_ of 5–10 mmHg and an inoculation of 1 × 10^5^ CFU bacteria (Group A_2_).

**Table 3:** **ΔP**_**RA **_**and IE outcomes**

**Experimental design**	**Group A (n = 45)**			**Group B (n = 45)**		
	**1 × 10**^**5**^ **CFU**			**1 × 10**^**4**^ **CFU**		
	**No-IE**	**Faint IE**	**Severe IE**	**No-IE**	**Faint IE**	**Severe IE**
ΔP_RA_ (0–5 mmHg)	12/15 (80%)	3/15 (20%)	0/15 (0%)	14/15 (93.3%)	1/15 (6.7%)	0/15 (0%)
ΔP_RA_ (5–10 mmHg)	0/15 (0%)	14/15 (93.3%)	1/15 (6.7%)	12/15 (80%)	3/15 (20%)	0/15 (0%)
ΔP_RA_ (10–15 mmHg)	0/15 (0%)	4/15 (26.7%)	11/15 (73.3%)	10/15 (66.7%)	5/15 (33.3%)	0/15 (0%)

*No-IE*: Did not develop IE by echocardiography or by histologic findings; *Faint IE*: Infectious endocarditis could confirmed by histologic findings but not visualized by echocardiography; *Severe IE*: Infectious endocarditis confirmed by both echocardiography and histologic findings. *LV*: Left ventricle; *RV*: Right ventricle; *V*_*TR*_: Peak velocity of tricuspid valve regurgitation; *ΔP*_*RA*_: Right atrial pressure before and just after valvular impairment was used to calculate alterations of right atrial pressure, indicated as ΔP_RA_.

## Discussion

IE animal models have been used to evaluate proposed changes in medical treatment. Cardiac valves are damaged and the animal inoculated with bacteria. The most primitive method was to damage cardiac valves by complex surgical procedures [21]. This modality was criticized because of the high mortality rate of the experimental animals. A catheter-related model for IE has been widely used since first introduced by Garrison and Freedoman [12] in 1970s. Usually, the catheter was used to destroy aortic valves when it was retrograded from carotid artery into ascending aorta [13]. However, it might cause relatively high mortality for the experimental animals. According to our experience, the heart rates of experimental animals such as rats or rabbits were very high. The retrograded catheter may severely damage aortic valves and then cause heart failure immediately. Many animals died within minutes after catheterization.

We developed a method [20] using a catheter system to damage the tricuspid valves under echocardiographic visualization. We reported the use of the catheter system with a guide wire inside to damage the tricuspid valves. We demonstrated a high survival rate and high infection rate with this reliable model. There were several limitations to this model. First, a high density of bacteria was used to ensure successful infection. This IE model was suited for therapeutic purposes, but not for prophylactic purposes. Second, the extent of tricuspid damage could not be quantitatively assessed when injuring the cardiac valves. Which animals were suitably infected could not be determined until several days after bacterial inoculation.

In the current study, we evaluated the use of different inoculum doses in the development of an IE model. Two doses of bacteria were inoculated. 1 × 10^5^ CFU was best suited for an IE prophylactic model. Previous studies have commonly used 1 × 10^6^ CFU [22] or even more [10-13]. The use of a relatively low density of inoculated bacteria is a highlight of this study.

Tricuspid valve regurgitation was confirmed in this new model just after damaging the tricuspid valves. There was not a difference in the V_TR_ of No-IE, Faint-IE, and Severe-IE animals. Inoculation density and valvular damage are the two main factors controlled in the development of an IE model. V_TR_ was not a sensitive predictor of valvular impairment or the successful creation an IE model. Differences in ΔP_RA_ were related to valvular impairment and IE. The value of ΔP_RA_ was useful in guiding valvular damage to make the IE model. A faint-IE model was reliably created when ΔP_RA_ was kept between 5–10 mmHg. The faint-IE model is relevant in assessing the impact of medications on early stage IE. This right-sided IE model simulates human invasive diagnostic and therapeutic procedures, such as right heart catheter angiography, atrial septal defect occlusion, placement of intracardiac pacemakers [23].

In the current study, 90 animals were used to create IE models. ΔP_RA_ and inoculation dosage were used to evaluate the technique. Echocardiographic and physiologic parameters were also evaluated. The relatively large number of experimental animals and the detailed experimental design were strengths of this study. We found that control of ΔP_RA_ during catheterization and tricuspid valve damage was associated with the successful creation of IE, another highlight of our study. Our method is different from previous reports [12,13] as the catheter system was removed immediately after catheterization. This modification is suitable for modeling intracardiac catheter procedures and the associated use of antibiotics [11,24].

Compared with traditional techniques, we proposed a novel method to create an IE model in rabbits. A relatively low bacterial innoculum made this model more suitable for a prophylaxis purpose. In addition, echocardiographic guidance made it possible for the catheter systems precisely damage the valve. The most important point, we demonstrated that ΔP_RA_ could precisely assess valvular impairment and then predict the success of the IE models during catheterization. It could save lots of experimental animals using this novel method. Limitation of the study is the time of the procedures. Repeated measurements of ΔP_RA_ could prolong the procedures. For our study, the mean time for catheterization and measurement of ΔP_RA_ was 25 ± 4 min.

At last, we could read some additional message from the current study. V_TR_ could not reflect the extent of valvular impairment just after injury, which indicates that V_TR_ is not a sensitive index to reflect ΔP_RA._ Also, one may imagine that it is not always reliable to estimate pulmonary artery pressure using V_TR._ For example, V_TR_ is very low (tricuspid regurgitation could hardly be detected) while the pulmonary artery pressure is above 50 mmHg measured by cardiac catheterization for a newborn.

## Conclusion

We described a method to create a faint IE model using a relatively low bacterial inoculum. ΔP_RA_ was used to assess valvular impairment. Controlling the value of ΔP_RA_ during catheterization and inoculating of an appropriate dose of bacteria was associated with a successful IE model.

## Abbreviations

CFU: Colony-forming units; IE: Infective endocarditis; LV: Left ventricle; R_PA_: Right atrial pressure; ΔR_PA_: Alterations of right atrial pressure; RV: Right ventricle; V_TR_: Peak velocity of tricuspid valve regurgitation.

## Competing interests

The authors declare that they have no competing interests.

## Authors’ contributions

M-lW and YZ designed the whole study. M-lW, YZ, MF, Y-jG, W-dR, and E-jL drafted the manuscript. YZ, Y-jG and M-lW performed right heart catheterization, echocardiographic examinations, and physiologic examinations, respectively. M-lW performed the microbiologic experiments. YZ and M-lW interpreted the results and analysed the data. All authors have read and approved the final manuscript.
